# Concise Synthesis of Functionalized Cyclobutene Analogues for Bioorthogonal Tetrazine Ligation

**DOI:** 10.3390/molecules26020276

**Published:** 2021-01-08

**Authors:** Jiayu Sun, Jie Li, Hongbao Sun, Chunling Li, Haoxing Wu

**Affiliations:** 1 Department of Radiology, West China Hospital, Sichuan University, Guo Xue Xiang 37, Chengdu 610041, China; sjy080512@163.com (J.S.); lj_jievip@163.com (J.L.); hbsun07@sina.com (H.S.); 2 Department of Neurosurgery, Sichuan Provincial People’s Hospital, University of Electronic Science and Technology of China, Chengdu 610072, China; 3 Chinese Academy of Sciences Sichuan Translational Medicine Research Hospital, Chengdu 610072, China

**Keywords:** functionalized cyclobutene analogues, concise synthesis, tetrazine ligation, bioorthogonal chemistry

## Abstract

Novel bioorthogonal tools enable the development of new biomedical applications. Here we report the concise synthesis of a series of aryl-functionalized cyclobutene analogues using commercially available starting materials. Our study demonstrates that cyclobutene acts as a small, strained dienophile to generate stable substrates suitable for bioorthogonal tetrazine ligation.

## 1. Introduction

Tetrazine has attracted increasing attention since the emergence of bioorthogonal chemistry [1,2,3,4]. Recent clinical trials have highlighted the biomedical usefulness of the bioorthogonal reaction between tetrazine and dienophiles, an inverse electron demand Diels–Alder (IEDDA) reaction [5,6,7,8]. A wide range of dienophiles, each with different advantages and limitations, have been developed as IEDDA substrates for various research purposes. In particular, highly strained trans-cyclooctenes (TCO) can react with tetrazine very rapidly, enabling a low concentration reaction in vivo in a short time [9,10,11]. The reaction of allyl-substituted TCO with tetrazine leads to a cascade elimination that has been widely used in the design of prodrugs [12,13,14,15]. Bicyclooctynes (BCN) react rapidly with tetrazine to afford pyridazine as a single product, which can facilitate downstream fluorogenic probe design [16,17,18]. Cyclopropene units have also emerged as alternative, more compact tags that can react quickly with tetrazine and have proven useful in studies of metabolic engineering [19,20,21,22]. The strained dienophiles norbornenes and norbornadienes, although they react less quickly with tetrazine, serve as a handle for covalent biomolecule labeling or offer a click-to-release feature that can be exploited for template-promoted turnover amplification, allowing the detection of endogenous oncogenic microRNAs [23,24,25,26,27]. In addition, the vinyl ether group is small and easy to prepare and can be decorated onto polymers and fluorophores under mild conditions [28,29,30,31,32]. Therefore, the development of novel dienophiles with different features is expected to provide straightforward access to new applications.

Cyclobutenes, as small and strained dienophiles, may be good mini-tags for labeling proteins, since their compactness makes it unlikely that they will disturb the proteins’ physiological functions. In addition, cyclobutene is less volatile than cyclopropene, which makes the synthesis of mini-tags easier. Despite the advantages of cyclobutene, only the synthesis and bioorthogonal properties of alkyl-substituted cyclobutene analogues have been investigated so far [33].

In this study, we report the concise synthesis of a series of aryl-substituted cyclobutene derivatives from commercially available 1,3-cyclobutanediol derivatives. We also systematically investigated the stability of the derivatives and their kinetics in bioorthogonal reactions.

## 2. Results and Discussion

**Scheme 1:**
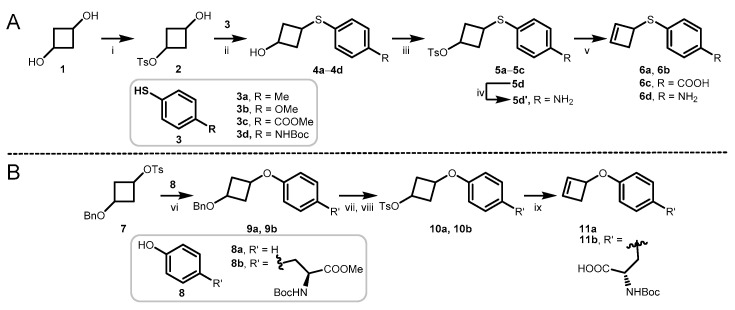
Synthesis of the cyclobutene derivatives. (**A**) Synthesis of phenylthio cyclobutene derivatives **6a**–**6d**; (**B**) Synthesis of phenolic cyclobutene derivatives **11a** and **11b**. (i) TsCl, Et_3_N, DCM, r.t., 9 h; (ii) *^t^*BuOK, *^t^*BuOH, 80 °C, 7 h, 32–86%; (iii) TsCl, Et_3_N, DCM, r.t., overnight, 55–79%; (iv) TFA, DCM, r.t., 1 h; (v) *^t^*BuOK, DMSO, r.t., 1 h, 46–89%; (vi) Cs_2_CO_3_, DMF, 80 °C, 8 h, 48–70%; (vii) Pd/C, EtOH, H_2_, r.t., overnight; (viii) Et_3_N, TsCl, DCM, r.t., 20 h, 70–86% for two steps; (ix) *^t^*BuOK, DMSO, r.t., 1 h, 45–57%. TsCl: 4-tosyl chloride; TFA: trifluoroacetic acid.

We used 1,3-cyclobutanediol (**1**) as the starting material for the synthesis of the desired cyclobutene derivatives, and we assumed that the two hydroxyl groups would be directly converted into a double bond or functional groups upon reaction under appropriate conditions. Treating 1,3-cyclobutanediol with *p*-toluenesulfonyl chloride (TsCl) and triethylamine (TEA) yielded the mono-toluene-4-sulfonate ester **2** in moderate yield. After flash column purification, the sulfonate ester reacted with a series of *p*-substituted thiophenols (**3a**–**3d**), forming the corresponding thioethers **4a**–**4d** in up to 86% yield. Subsequent tosylation and elimination generated four novel cyclobutene derivatives (**6a**–**6d**) bearing a phenyl sulfide moiety useful for further functionalization (Scheme 1A). Phenolic cyclobutene derivatives were prepared by slightly modifying the above mentioned steps and by using the commercially available 3-(benzyloxy)cyclobutyl-4-methylbenzenesulfonate (**7**). We followed a three-step process (Scheme 1B) to prepare two cyclobutene analogues (**11a** and **11b**) in overall yield of 22–23.5%. None of the intermediates or final products were volatile, and all were stored for months at −20 °C.

**Figure 1:**
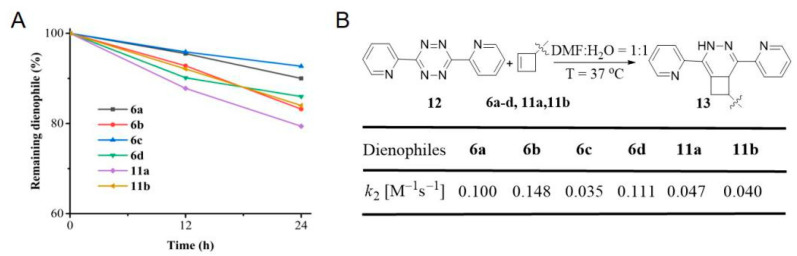
(**A**) Stability of the cyclobutene derivatives **6a**–**6d**, **11a**, and **11b** during incubation in *N,N*-dimethylformamide/phosphate-buffered saline (1:1) at 37 °C for 24 h. Absorption was monitored using high-performance liquid chromatography. (**B**) Kinetics of the inverse electron demand Diels–Alder (IEDDA) reactions between tetrazine **12** and **6a**–**6d**, **11a**, and **11b**. Conditions: 0.5 mM tetrazine, 5 mM dienophile, DMF/H_2_O (1:1), 37 °C.

The synthesized cyclobutene derivatives were then incubated in phosphate-buffered saline (PBS) with 50% *N*,*N*-dimethylformamide (DMF) as co-solvent at 37 °C, and their stability was monitored over time using liquid chromatography–mass spectrometry. All cyclobutenes were relatively stable under these buffer conditions, irrespective of the phenyl substituent. After incubation for 12 h, 90% of the original analogues were still present, except for compound **11a** (87%) (Figure 1A). There was slight degradation for all analogues, with 79.4–92.7% of the original material remaining at 24 h. These aryl-substituted cyclobutenes are slightly less stable than some other known dienophiles, such as TCO [9], cyclopropene units [19,34], and norbornene derivatives [23,26]. However, they are facilely prepared, so they could be a choice in the toolbox for preliminary studies.

**Figure 2:**
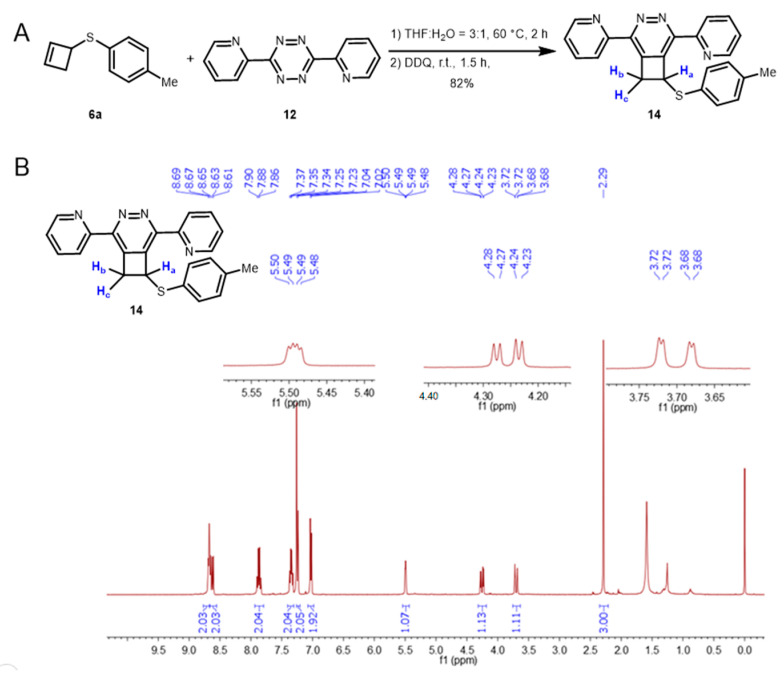
(**A**) IEDDA-oxidation cascade reaction of cyclobutene **6a** with dipyridinetetrazine **12**. (**B**) The ^1^H–NMR spectrum of compound **14**.

Next, we reacted the six cyclobutene analogues **6a**–**6d**, **11a**, and **11b** with *di*-pyridyl-tetrazine under pseudo-first-order conditions (Figure 1B). The reactants were dissolved in 50% DMF in PBS, and the reaction at 37 °C was monitored using UV–vis spectroscopy to track the disappearance of the characteristic absorption peak of tetrazine at 520 nm over time. The IEDDA reaction between the cyclobutenes and tetrazine proceeded with rate constants of 0.04–0.15 M^−1^ s^−1^, similar to those of *N*-acylazetines with tetrazine [35,36], and faster than those of terminal alkenes with tetrazine [37]. Electron-donating substituents on the phenyl ring of the cyclobutene analogues improved their reactivity, while a benzoic acid group slightly reduced the reactivity of the double bond. In order to examine the structure of the IEDDA product, the dihydropyridazine intermediate between **6a** and **12** was further oxidized using 1.1 equivalent of 2,3-dichloro-5,6-dicyano-1,4-benzoquinone (DDQ), affording a single pyridazine product **14** as the major product in 82% overall yield. The structure of **14** was confirmed by nuclear magnetic resonance (NMR) spectroscopy (Figure 2) which indicated that almost no ring-opening or sideproduct formation had occurred in the two-step reactions.

**Scheme 2:**
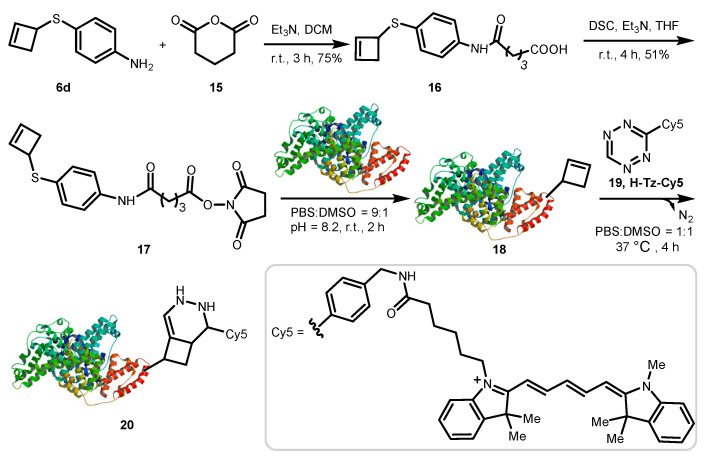
In vitro cyclobutene modification and bioorthogonal labeling of protein bovine serum albumin (BSA).

**Figure 3:**
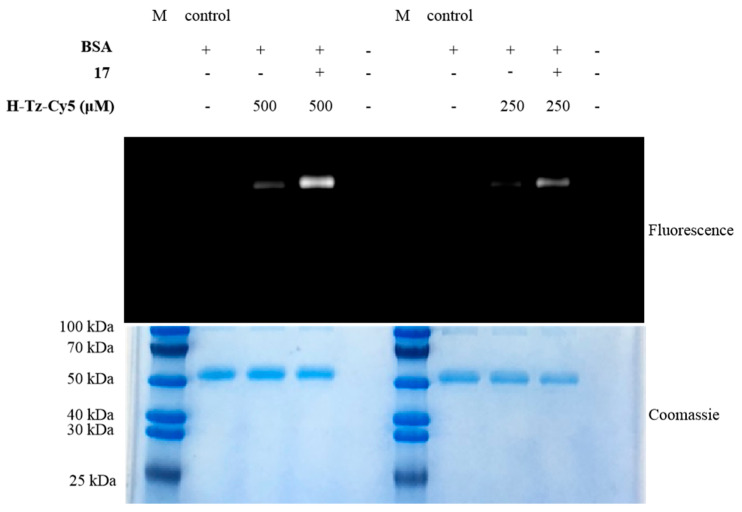
In vitro labeling of bovine serum albumin (BSA) via cyclobutene–tetrazine bioorthogonal reaction. *Upper panel*: fluorescent image of the same gel before Coomassie blue staining. *Lower panel*: Coomassie-blue-stained gel of BSA modified through IEDDA reaction between conjugate **17** and the Cy5-containing fluorescent probe **19** (250 μM or 500 μM). Control reactions contained only BSA or BSA with only the Cy5 probe (250 μM or 500 μM). “M”: protein marker.

To examine the applicability of our cyclobutenes for biomolecular labeling, we first synthesized a cyclobutene–*N*-hydroxysuccinimide (NHS) amide (**17**) using the cyclobutene derivative **6d** via a two-step process (Scheme 2). As a proof of concept, the cyclobutene–NHS conjugate **17** was added to bovine serum albumin (BSA) to create a cyclobutene “handle” for bioorthogonal labeling. The modified BSA was incubated with a tetrazine-Cy5 near-infrared probe (**19**, H-Tz-Cy5) for 4 h. Subsequent analysis with sodium dodecyl sulfate–polyacrylamide gel electrophoresis showed that the cyclobutene-modified BSA was fluorescent, but the control reactions lacking the conjugate **17** or the Cy5 probe were not (Figure 3). A rough estimation of the yield of the labeling reaction on the model BSA protein was calculated to be about 29.1%. These results suggest that cyclobutene derivatives can be used to label proteins via tetrazine bioorthogonal reactions.

## 3. Experimental Section

### 3.1. Materials

All chemical reagents were purchased from commercial sources (Adamas-Beta, Shanghai, China; Energy Chemical, Shanghai, China; or Sigma-Aldrich, St. Louis, MO, USA) and used without further purification. The silica gel plates were purchased from Yantai Jiangyou Silica Gel Development (silica 200 × 200 mm, pH 6.2–6.8, glass-backed). All reactions were monitored based on absorbance at 254 nm and liquid chromatography–mass spectrometry (Agilent-Technologies Infinity Lab LC/MSD system, Agilent Technologies Inc., Santa Clara, CA, USA). Products were purified by flash silica gel column chromatography (SepaBeanTM, Santai Technologies, Changzhou, Jiangsu, China). ^1^H– and ^13^C–NMR spectra were recorded on a Bruker (Billerica, MA, USA) NMR spectrometer (^1^H, 400 MHz; ^13^C, 101 MHz) and high-resolution mass spectrometry (Bruker, Billerica, MA, USA) was performed on a Bruker Daltonics Data analysis 3.4 system. Reaction kinetics were determined on a Quawell Scientific Q6000+ microvolume spectrophotometer. Protein gels were analyzed for Cy5 fluorescence using the ChemiDoc™ MP imaging system (Bio-Rad Laboratories Inc., Hercules, CA, USA).

### 3.2. Synthesis and Characterization

#### 3.2.1. Synthesis of 3-Hydroxycyclobutyl 4-Methylbenzenesulfonate (**2**)

A solution of tosyl chloride (TsCl, 22 mg, 0.12 mmol, 0.7 equiv) in dichloromethane (DCM, 0.5 mL) was added dropwise with stirring to a mixture of 1,3-cyclobutanediol (15 mg, 0.17 mmol, 1.0 equiv) and TEA (47 μL, 0.34 mmol, 2.0 equiv) in DCM (0.3 mL) at 0 °C under Ar. The reaction mixture was stirred at room temperature for 9 h, followed by evaporation in vacuo. The obtained residue was purified by silica gel column chromatography (PE/EA = 2:1) to afford the desired analogue **2** in 38% yield as a light yellow oil.

#### 3.2.2. Synthesis of Compounds **4a**–**4d**

To synthesize 3-(*p*-tolylthio)cyclobutanol (**4a**), potassium *tert*-butoxide (265 mg, 2.35 mmol, 1.3 equiv) was added under intense stirring to a solution of 3-hydroxycyclobutyl-4-methylbenzenesulfonate (439 mg, 1.81 mmol, 1.0 equiv) and 4-methylbenzenethiol (270 mg, 2.18 mmol, 1.2 equiv) in *tert*-butanol (8 mL) at room temperature under Ar. The mixture was stirred at 80 °C for 7 h. After completion of the reaction monitored by TLC, the crude mixture was quenched with water and extracted with ethyl acetate. The combined organic phases were washed with brine, dried over anhydrous Na_2_SO_4_, and evaporated in vacuo. The residue was purified by silica gel column chromatography (PE/EA = 3:1) to afford **4a** (186 mg, 86%) as a yellow solid. ^1^H–NMR (400 MHz, CDCl_3_) *δ* 7.16 (*d*, *J* = 8.1 Hz, 2H), 7.10 (*d*, *J* = 8.0 Hz, 2H), 4.63–4.56 (*m*, 1H), 3.85–3.79 (*m*, 1H), 2.46–2.34 (*m*, 4H), 2.32 (*s*, 3H), 1.99 (*s*, 1H). ^13^C–NMR (101 MHz, CDCl_3_) *δ* 136.2, 132.9, 129.8, 129.7, 66.3, 39.6, 33.9, 21.1. HRMS [M+H]^+^ *m*/*z* calcd. for [C_11_H_15_OS]^+^ 195.0838, found 195.0837.

To synthesize 3-((4-methoxyphenyl)thio)cyclobutanol (**4b**), potassium *tert*-butoxide (105 mg, 0.94 mmol, 1.3 equiv) was added under intense stirring to a solution of 3-hydroxycyclobutyl-4-methylbenzenesulfonate (170.3 mg, 0.72 mmol, 1.0 equiv) and 4-methoxybenzenethiol (121 mg, 0.86 mmol, 1.2 equiv) in *tert*-butanol (4 mL) at room temperature under Ar. The mixture was stirred at 80 °C for 7 h. After completion of the reaction monitored by TLC, the crude mixture was quenched with water and extracted with ethyl acetate. The combined organic phases were washed with brine, dried over anhydrous Na_2_SO_4_, and evaporated in vacuo. The residue was purified by silica gel column chromatography (PE/EA = 20:1) to afford **4b** (81 mg, 77%) as a white solid. ^1^H–NMR (400 MHz, CDCl_3_) δ 7.31–7.26 (m, 2H), 6.87–6.81 (m, 2H), 4.58–4.52 (*m*, 1H), 3.79 (s, 3H), 3.77–3.69 (m, 1H), 2.34 (t, *J* = 6.4 Hz, 4H), 1.90 (s, 1H). ^13^C–NMR (101 MHz, CDCl_3_) δ 159.1, 133.2, 126.4, 114.7, 66.2, 55.4, 39.5, 35.2.

To synthesize methyl 4-((3-hydroxycyclobutyl)thio)benzoate (**4c**), potassium *tert*-butoxide (304 mg, 2.70 mmol, 1.3 equiv) was added under intense stirring to a solution of 3-hydroxycyclobutyl-4-methylbenzenesulfonate (504 mg, 2.08 mmol, 1.0 equiv) and methyl 4-mercaptobenzoate (420 mg, 2.50 mmol, 1.2 equiv) in *tert*-butanol (8 mL) at room temperature under Ar. The mixture was stirred at 80 °C for 7 h. After completion of the reaction monitored by TLC, the crude mixture was quenched with water and extracted with ethyl acetate. The combined organic phases were washed with brine, dried over anhydrous Na_2_SO_4_, and evaporated in vacuo. The residue was purified by silica gel column chromatography (PE/EA = 2:1) to afford **4c** (200 mg, 48%) as a white solid. ^1^H–NMR (400 MHz, CDCl_3_) δ 7.91 (d, *J* = 8.3 Hz, 2H), 7.15 (d, *J* = 8.3 Hz, 2H), 4.75–4.57 (m, 1H), 3.98–3.92 (*m*, 1H), 3.89 (s, 3H), 2.60–2.48 (m, 2H), 2.43–2.37 (*m*, 2H), 2.13 (s, 1H). ^13^C–NMR (101 MHz, CDCl_3_) δ 167.0, 144.5, 130.1, 126.7, 126.0, 66.1, 52.2, 52.1, 39.4, 31.9. HRMS [M+Na]^+^ *m*/*z* calcd. for [C_12_H_14_NaO_3_S]^+^ 261.0556, found 261.0563.

To synthesize tert-butyl (4-((3-hydroxycyclobutyl)thio)phenyl)carbamate (**4d**), potassium *tert*-butoxide (200 mg, 1.78 mmol, 1.3 equiv) was added under intense stirring to a solution of 3-hydroxycyclobutyl-4-methylbenzenesulfonate (331 mg, 1.36 mmol, 1.0 equiv) and methyl tert-butyl (4-mercaptophenyl)carbamate (370 mg, 1.64 mmol, 1.2 equiv) in *tert*-butanol (8 mL) at room temperature under Ar. The mixture was stirred at 80 °C for 7 h. After completion of the reaction monitored by TLC, the crude mixture was quenched with water and extracted with ethyl acetate. The combined organic phases were washed with brine, dried over anhydrous Na_2_SO_4_, and evaporated in vacuo. The residue was purified by silica gel column chromatography (PE/EA = 2:1) to afford **4d** (146 mg, 32%) as a brown solid. ^1^H–NMR (400 MHz, CDCl_3_) δ 7.29 (d, *J* = 8.4 Hz, 2H), 7.22 (d, *J* = 8.6 Hz, 2H), 6.48 (s, 1H), 4.63–4.49 (m, 1H), 3.82–3.72 (m, 1H), 2.36 (dd, *J* = 12.8, 6.4 Hz, 4H), 1.86 (s, 1H), 1.51 (s, 9H). ^13^C–NMR (101 MHz, CDCl_3_) δ 152.7, 137.2, 131.4, 129.7, 119.2, 80.8, 66.2, 39.5, 34.5, 28.4. HRMS [M-H]^−^ *m*/*z* calcd. for [C_15_H_20_NO_3_S]^−^ 294.1169, found 294.1167.

#### 3.2.3. Synthesis of Compounds **5a**–**5d**

To synthesize 3-(*p*-Tolylthio)cyclobutyl-4-methylbenzenesulfonate (**5a**), a solution of TsCl (365 mg, 1.91 mmol, 2.0 equiv) in DCM (5 mL) was added dropwise under stirring to a mixture of 3-(*p*-tolylthio)cyclobutanol (186 mg, 0.96 mmol, 1.0 equiv), TEA (400 μL, 2.87 mmol, 3 equiv), and 4-dimethylaminopyridine (DMAP) (23 mg, 0.19 mmol, 0.2 equiv) in DCM (5 mL) at 0 °C under Ar. The resulting mixture was stirred at room temperature overnight. After completion, the reaction was quenched with water and extracted with ethyl acetate. The combined organic phases were washed with brine, dried over anhydrous Na_2_SO_4_, and evaporated in vacuo. The residue was purified by silica gel column chromatography (PE/EA = 2:1) to afford **5a** (261 mg, 78%) as a light yellow oil. ^1^H–NMR (400 MHz, CDCl_3_) *δ* 7.76 (*d*, *J* = 8.3 Hz, 2H), 7.33 (*d*, *J* = 8.0 Hz, 2H), 7.11 (*q*, *J* = 8.3 Hz, 4H), 5.03–4.96 (*m*, 1H), 3.84–3.73 (*m*, 1H), 2.70–2.56 (*m*, 2H), 2.45 (*s*, 3H), 2.36–2.26 (*m*, 5H). ^13^C–NMR (101 MHz, CDCl_3_) *δ* 145.0, 136.9, 133.8, 131.7, 130.4, 130.0, 129.9, 128.0, 73.9, 37.2, 34.6, 21.8, 21.2. HRMS [M+Na]^+^ *m/z* calcd. for [C_18_H_20_NaO_3_S_2_]^+^ 371.0746, found 371.0749.

To synthesize 3-((4-methoxyphenyl)thio)cyclobutyl 4-methylbenzenesulfonate (**5b**), a solution of TsCl (90.7 mg, 0.48 mmol, 2.0 equiv) in DCM (2 mL) was added dropwise under stirring to a mixture of 3-((4-methoxyphenyl)thio)cyclobutanol (50 mg, 0.24 mmol, 1.0 equiv), triethylamine (TEA, 100 μL, 0.71 mmol, 3 equiv), and 4-dimethylaminopyridine (DMAP) (5.8 mg, 0.05 mmol, 0.2 equiv) in DCM (2 mL) at 0 °C under Ar. The resulting mixture was stirred at room temperature overnight. After completion, the reaction was quenched with water and extracted with ethyl acetate. The combined organic phases were washed with brine, dried over anhydrous Na_2_SO_4_, and evaporated in vacuo. The residue was purified by silica gel column chromatography (PE/EA = 5:1) to afford **5b** (47.6 mg, 55%) as a white solid. ^1^H–NMR (400 MHz, CDCl_3_) δ 7.75 (d, *J* = 8.3 Hz, 2H), 7.33 (d, *J* = 8.1 Hz, 2H), 7.25 (d, *J* = 8.9 Hz, 2H), 6.83 (d, *J* = 8.8 Hz, 2H), 4.97–4.90 (*m*, 1H), 3.79 (s, 3H), 3.74–3.67 (*m*, 1H), 2.63–2.51 (m, 2H), 2.44 (s, 3H), 2.30–2.25 (*m*, 2H). ^13^C–NMR (101 MHz, CDCl_3_) δ 159.5, 145.0, 133.9, 133.7, 130.0, 127.9, 125.2, 114.8, 73.9, 55.4, 37.0, 35.8, 21.7. HRMS [M+Na]^+^ *m*/*z* calcd. for [C_18_H_20_NaO_4_S_2_]^+^ 387.0695, found 387.0700.

To synthesize methyl 4-((3-(tosyloxy)cyclobutyl)thio)benzoate (**5c**), a solution of TsCl (318.8 mg, 1.67 mmol, 2.0 equiv) in DCM (3 mL) was added dropwise under stirring to a mixture of methyl 4-((3-hydroxycyclobutyl)thio)benzoate (199 mg, 0.84 mmol, 1.0 equiv), TEA (350 μL, 2.5 mmol, 3 equiv), and 4-dimethylaminopyridine (DMAP) (20.4 mg, 0.16 mmol, 0.2 equiv) in DCM (4 mL) at 0 °C under Ar. The resulting mixture was stirred at room temperature overnight. After completion, the reaction was quenched with water and extracted with ethyl acetate. The combined organic phases were washed with brine, dried over anhydrous Na_2_SO_4_, and evaporated in vacuo. The residue was purified by silica gel column chromatography (PE/EA = 5:1) to afford **5c** (260 mg, 79%) as a white solid. ^1^H–NMR (400 MHz, CDCl_3_) δ 7.91 (d, *J* = 8.6 Hz, 2H), 7.77 (d, *J* = 8.3 Hz, 2H), 7.34 (d, *J* = 8.0 Hz, 2H), 7.11 (d, *J* = 8.5 Hz, 2H), 5.09–5.02 (*m*, 1H), 3.98–3.93 (*m*, 1H), 3.89 (s, 3H), 2.85–2.69 (m, 2H), 2.45 (s, 3H), 2.40–2.33 (*m*, 2H). ^13^C–NMR (101 MHz, CDCl_3_) δ 166.7, 145.1, 143.2, 133.6, 130.2, 130.0, 127.9, 127.2, 126.4, 73.4, 52.2, 37.0, 32.5, 21.8. HRMS [M+Na]^+^ *m/z* calcd. for [C_19_H_20_NaO_5_S_2_]^+^ 415.0644, found 415.0652.

To synthesize 3-((4-((tert-butoxycarbonyl)amino)phenyl)thio)cyclobutyl 4-methylbenzene- sulfonate (**5d**), a solution of TsCl (155 mg, 0.81 mmol, 2.0 equiv) in DCM (2 mL) was added dropwise under stirring to a mixture of tert-butyl (4-((3-hydroxycyclobutyl)thio)phenyl)carbamate (120 mg, 0.41 mmol, 1.0 equiv), TEA (170 μL, 1.22 mmol, 3 equiv), and 4-dimethylaminopyridine (DMAP) (10 mg, 0.08 mmol, 0.2 equiv) in DCM (2 mL) at 0 °C under Ar. The resulting mixture was stirred at room temperature overnight. After completion, the reaction was quenched with water and extracted with ethyl acetate. The combined organic phases were washed with brine, dried over anhydrous Na_2_SO_4_, and evaporated in vacuo. The residue was purified by silica gel column chromatography (PE/EA = 5:1) to afford **5d** (136 mg, 74%) as a light yellow oil. ^1^H–NMR (400 MHz, CDCl_3_) δ 7.75 (d, *J* = 8.3 Hz, 2H), 7.31 (dd, *J* = 15.9, 8.4 Hz, 4H), 7.19 (d, *J* = 8.6 Hz, 2H), 6.51 (s, 1H), 5.04–4.89 (m, 1H), 3.77–3.71 (*m*, 1H), 2.64–2.52 (m, 2H), 2.44 (s, 3H), 2.31–2.25 (*m*, 2H), 1.51 (s, 9H). ^13^C–NMR (101 MHz, CDCl_3_) δ 152.7, 145.0, 137.8, 133.7, 132.2, 130.0, 128.3, 127.9, 119.2, 80.9, 73.9, 37.0, 35.1, 28.4, 21.8. HRMS [M-H]^−^ *m/z* calcd. for [C_22_H_26_NO_5_S_2_]^−^ 448.1258, found 448.1256.

#### 3.2.4. Synthesis of Compounds **6a**–**6d**

To synthesize cyclobut-2-en-1-yl(*p*-tolyl)sulfane (**6a**), potassium *tert*-butoxide (56 mg, 0.5 mmol, 2.0 equiv) was dissolved in dry DMSO (1 mL) under Ar, forming a colorless solution. Then, a solution of 3-(*p*-tolylthio)cyclobutyl-4-methylbenzenesulfonate (87 mg, 0.25 mmol, 1.0 equiv) in dry DMSO (l mL) was added slowly to the colorless solution, and the resulting mixture was stirred at room temperature for 1 h. Water was then added slowly, followed by ethyl acetate. The separated water phase was extracted with ethyl acetate three times, and the combined organic phases were washed once with water. The final organic phase was dried over anhydrous Na_2_SO_4_ and evaporated in vacuo. The residue was purified by silica gel column chromatography (PE) to afford compound **6a** (38 mg, 86%) as a light yellow oil. ^1^H–NMR (400 MHz, CDCl_3_): *δ* 7.25 (*d*, *J* = 8.0 Hz, 2H), 7.09 (*d*, *J* = 7.9 Hz, 2H), 6.10 (*dd*, *J* = 17.4, 2.5 Hz, 2H), 4.28 (*d*, *J* = 3.7 Hz, 1H), 3.03 (*dd*, *J* = 13.8, 3.9 Hz, 1H), 2.49 (*d*, *J* = 13.8 Hz, 1H), 2.31 (*s*, 3H). ^13^C–NMR (101 MHz, CDCl_3_): *δ* 138.1, 137.5, 136.5, 132.4, 130.7, 129.7, 46.9, 40.1, 21.2. HRMS [M+H]^+^ *m/z* calcd. for [C_11_H_13_S]^+^ 177.0732, found 177.0740.

To synthesize cyclobut-2-en-1-yl(4-methoxyphenyl)sulfane (**6b**), potassium *tert*-butoxide (29.3 mg, 0.26 mmol, 2.0 equiv) was dissolved in dry DMSO (1 mL) under Ar, forming a colorless solution. Then, a solution of 3-((4-methoxyphenyl)thio)cyclobutyl 4-methylbenzenesulfonate (47.6 mg, 0.13 mmol, 1.0 equiv) in dry DMSO (l mL) was added slowly to the colorless solution, and the resulting mixture was stirred at room temperature for 1 h. Water was then added slowly, followed by ethyl acetate. The separated water phase was extracted with ethyl acetate three times, and the combined organic phases were washed once with water. The final organic phase was dried over anhydrous Na_2_SO_4_ and evaporated in vacuo. The residue was purified by silica gel column chromatography (PE) to afford compound **6b** (24.5 mg, 89%) as a light yellow oil. ^1^H–NMR (400 MHz, CDCl_3_) δ 7.35 (d, *J* = 8.7 Hz, 2H), 6.84 (d, *J* = 8.7 Hz, 2H), 6.07 (dd, *J* = 14.4, 2.6 Hz, 2H), 4.19 (d, *J* = 3.9 Hz, 1H), 3.80 (s, 3H), 2.97 (dd, *J* = 13.8, 4.0 Hz, 1H), 2.46 (d, *J* = 13.8 Hz, 1H). ^13^C–NMR (101 MHz, CDCl_3_) δ 159.2, 138.3, 137.2, 134.1, 125.6, 114.4, 55.4, 47.9, 39.8. HRMS [M+H]^+^ *m/z* calcd. for [C_11_H_13_OS]^+^ 193.0682, found 193.0686.

To synthesize 4-(cyclobut-2-en-1-ylthio)benzoic acid (**6c**), potassium *tert*-butoxide (11.45 mg, 0.10 mmol, 2.0 equiv) was dissolved in dry DMSO (0.5 mL) under Ar, forming a colorless solution. Then, a solution of methyl 4-((3-(tosyloxy)cyclobutyl)thio)benzoate (20 mg, 0.05 mmol, 1.0 equiv) in dry DMSO (0.5 mL) was added slowly to the colorless solution, and the resulting mixture was stirred at room temperature for 1 h. Water was then added slowly, followed by ethyl acetate. The separated water phase was extracted with ethyl acetate three times, and the combined organic phases were washed once with water. The final organic phase was dried over anhydrous Na_2_SO_4_ and evaporated in vacuo. The residue was purified by silica gel column chromatography (PE/EA = 1:1) to afford compound **6c** (5.4 mg, 47%) as a white solid. ^1^H–NMR (400 MHz, DMSO) δ 7.85 (d, *J* = 8.4 Hz, 2H), 7.34 (d, *J* = 8.4 Hz, 2H), 6.25 (d, *J* = 24.8 Hz, 2H), 4.57 (s, 1H), 3.19 (dd, *J* = 13.9, 3.8 Hz, 1H), 2.43 (d, *J* = 14.0 Hz, 1H). ^13^C–NMR (101 MHz, DMSO) δ 167.0, 143.3, 138.3, 137.2, 129.8, 129.1, 126.2, 43.8, 39.6. HRMS [M-H]^−^ *m/z* calcd. for [C_11_H_9_O_2_S]^−^ 205.0329, found 205.0330.

To synthesize 4-(cyclobut-2-en-1-ylthio)aniline (**6d**), 3-((4-((tert-butoxycarbonyl)amino)phenyl) thio)cyclobutyl 4-methylbenzenesulfonate (22 mg, 0.049 mmol, 1.0 equiv) was dissolved in DCM (0.7 mL) and trifluoroacetic acid was added (0.3 mL). The resulting mixture was stirred at room temperature for 1 h. Upon completion monitored by TLC, the reaction mixture was evaporated under reduced pressure. The residue **5d’** was used for the next step without further purification. Potassium *tert*-butoxide (11 mg, 0.097 mmol, 2.0 equiv) was dissolved in dry DMSO (0.4 mL) under Ar, forming a colorless solution. Then, a solution of methyl 3-((4-aminophenyl)thio)cyclobutyl 4-methylbenzenesulfonate (17.1 mg, 0.049 mmol, 1.0 equiv) in dry DMSO (0.4 mL) was added slowly to the colorless solution, and the resulting mixture was stirred at room temperature for 1 h. Water was then added slowly, followed by ethyl acetate. The separated water phase was extracted with ethyl acetate three times, and the combined organic phases were washed once with water. The final organic phase was dried over anhydrous Na_2_SO_4_ and evaporated in vacuo. The residue was purified by silica gel column chromatography (PE/EA = 5:1) to afford compound **6d** (4 mg, 46%) as a light yellow solid. ^1^H–NMR (400 MHz, CDCl_3_) δ 7.25 (d, *J* = 9.3 Hz, 3H), 6.61 (d, *J* = 8.5 Hz, 2H), 6.05 (dd, *J* = 10.9, 2.6 Hz, 2H), 4.13 (d, *J* = 3.9 Hz, 1H), 3.73 (s, 2H), 2.93 (dd, *J* = 13.8, 4.0 Hz, 1H), 2.44 (d, *J* = 13.7 Hz, 1H). ^13^C–NMR (101 MHz, CDCl_3_) δ 146.2, 138.5, 137.0, 134.8, 122.2, 115.4, 48.3, 39.6. HRMS [M+H]^+^ *m/z* calcd. for [C_10_H_12_NS]^+^ 178.0685, found 178.0687.

#### 3.2.5. Synthesis of Compounds **9a** and **9b**

A solution of 3-(benzyloxy)cyclobutyl-4-methylbenzenesulfonate (1.0 equiv), compound **8a** or **8b** (2.0 equiv), and Cs_2_CO_3_ (2.0 equiv) was prepared in DMF, and the resulting mixture was stirred at 80 °C for 8 h. Then the reaction was quenched with water and extracted with ethyl acetate. The combined organic phases were washed with brine, dried over anhydrous Na_2_SO_4_, and evaporated in vacuo. The residue was purified by silica gel column chromatography (PE/EA = 40:1) to afford the desired product **9a** or **9b**.

For the synthesis of (3-(benzyloxy)cyclobutoxy)benzene (**9a**), 1.32 g of **8a** was used, giving 484 mg of compound **9a** (48% yield) as a light yellow oil.

For the synthesis of methyl 3-(4-(3-(benzyloxy)cyclobutoxy)phenyl) -2-((tert-butoxycarbonyl)amino)propanoate (**9b**), 261 mg of **8b** was used, giving 250 mg of compound **9b** (70% yield) as a white solid.

#### 3.2.6. Synthesis of Compounds **10a** and **10b**

To prepare 3-phenoxycyclobutyl-4-methylbenzenesulfonate (**10a**), compound **9a** (469 mg, 1.84 mmol, 1.0 equiv) was dissolved in ethanol (10 mL), followed by the addition of Pd/C (94 mg). The resulting solution was purged with hydrogen five times, and the mixture was then hydrogenated with a H_2_ balloon overnight at room temperature. After completion of the reaction monitored by TLC, the catalyst was removed by filtration over celite, and the filter cake was washed three times with ethyl acetate. The combined organic layers were evaporated in vacuo, affording a crude mixture that was used in the next step without purification. TEA (772 μL, 5.53 mmol, 3.0 equiv) and DMAP (45 mg, 0.37 mmol, 0.2 equiv) were dissolved in DCM (4 mL) under Ar and added to the crude mixture. The resulting mixture was cooled to 0 °C, and a solution of TsCl (1.05 g, 5.53 mmol, 3.0 equiv) in DCM (6 mL) was added. The reaction mixture was stirred at room temperature for 20 h. Then the reaction was quenched with water and extracted with ethyl acetate. The combined organic phases were washed with brine, dried over anhydrous Na_2_SO_4_, and evaporated in vacuo. The residue was purified by silica gel column chromatography (PE/EA = 5:1) to afford **10a** in 86% yield (508 mg) as a light yellow oil. ^1^H–NMR (400 MHz, CDCl_3_) *δ* 7.79 (*d*, *J* = 8.2 Hz, 2H), 7.35 (*d*, *J* = 8.3 Hz, 2H), 7.25 (*t*, *J* = 8.0 Hz, 2H), 6.94 (*t*, *J* = 7.4 Hz, 1H), 6.71 (*d*, *J* = 7.9 Hz, 2H), 5.11–5.00 (*m*, 1H), 4.84–4.79 (*m*, 1H), 2.67–2.58 (*m*, 2H), 2.54–2.48 (*m*, 2H), 2.45 (*s*, 3H). ^13^C–NMR (101 MHz, CDCl_3_) *δ* 157.2, 145.1, 133.7, 130.1, 129.7, 128.0, 121.3, 114.9, 73.2, 68.2, 37.8, 21.8. HRMS [M+Na]^+^ *m/z* calcd. for[C_17_H_18_NaO_4_S]^+^ 341.0818, found 341.0826.

Methyl 2-((tert-butoxycarbonyl)amino)-3-(4-(3-(tosyloxy)cyclobutoxy)phenyl)propanoate (**10b**) was isolated in 70% yield from compound **9b** and TsCl as a light red solid by a method analogous to that described for the synthesis of **10a** above.

#### 3.2.7. Synthesis of Compounds **11a** and **11b**

To prepare cyclobut-2-en-1-yloxy)benzene (**11a**), potassium *tert*-butoxide (198 mg, 1.77 mmol, 2.0 equiv) was dissolved in dry DMSO (2 mL) under Ar, forming a colorless solution. A solution of compound **10a** (281 mg, 0.88 mmol, 1.0 equiv) in dry DMSO (2 mL) was then added slowly to the colorless solution, and the reaction mixture was left stirring at room temperature for 1 h. After completion of the reaction monitored by TLC, water was added slowly, followed by ethyl acetate. The separated water phase was extracted with ethyl acetate three times, and the combined organic layers were washed once with water. The final organic phase was dried over anhydrous Na_2_SO_4_ and evaporated in vacuo. The residue was purified by silica gel column chromatography (PE) to afford **11a** (73.6 mg, 57%) as a colorless oil. ^1^H–NMR (400 MHz, CDCl_3_) *δ* 7.28 (*t*, *J* = 9.8 Hz, 2H), 6.95 (*t*, *J* = 7.4 Hz, 1H), 6.89 (*d*, *J* = 7.8 Hz, 2H), 6.31 (*d*, *J* = 33.7 Hz, 2H), 5.10 (*d*, *J* = 3.2 Hz, 1H), 3.00 (*dd*, *J* = 13.4, 3.5 Hz, 1H), 2.63 (*dd*, *J* = 13.4, 1.0 Hz, 1H). ^13^C–NMR (101 MHz, CDCl_3_) *δ* 158.2, 139.3, 137.0, 129.7, 121.0, 115.0, 74.5, 39.7.

The compound 2-((*tert*-butoxycarbonyl)amino)-3-(4-(cyclobut-2-en-1-yloxy)phenyl)propanoic acid (**11b**) was obtained in 45% yield as a white solid from compound **10b** and potassium *tert*-butoxide by a method analogous to that described for the synthesis of **11a** above. ^1^H–NMR (400 MHz, DMSO-*d*_6_) *δ* 12.49 (*s*, 1H), 7.11 (*d*, *J* = 8.4 Hz, 2H), 6.98 (*d*, *J* = 8.3 Hz, 1H), 6.77 (*d*, *J* = 8.5 Hz, 2H), 6.30 (*d*, *J* = 32.6 Hz, 2H), 5.02 (*d*, *J* = 2.8 Hz, 1H), 4.02–3.96 (*m*, 1H), 2.98–2.84 (*m*, 2H), 2.71 (*dd*, *J* = 13.6, 10.3 Hz, 1H), 2.48–2.35 (*m*, 2H), 1.28 (*s*, 8H). ^13^C–NMR (101 MHz, DMSO-*d*_6_) *δ* 173.7, 156.2, 155.5, 138.9, 137.2, 130.2, 130.1, 114.4, 78.0, 73.8, 55.4, 40.4, 35.7, 28.2, 27.9. HRMS [M-H]^−^ *m/z* calcd. for [C_18_H_22_NO_5_]^−^ 332.1503, found 332.1493.

#### 3.2.8. Synthesis of 5-((4-(Cyclobut-2-en-1-Ylthio)Phenyl)Amino)-5-Oxopentanoic Acid (**16**)

A mixture of compound **6d** (5 mg, 0.028 mmol, 1.0 equiv), glutaric anhydride (3.5 mg, 0.031 mmol, 1.1 equiv), and TEA (10 μL, 0.07 mmol, 2.5 equiv) was dissolved in DCM (0.5 mL) under Ar. The resulting mixture was stirred at room temperature for 3 h, and then quenched with water and extracted with ethyl acetate. The combined organic phases were washed with brine, dried over anhydrous Na_2_SO_4_, and evaporated in vacuo. Due to the small scale of the reaction, the residue was purified by thin-layer chromatography to afford 6.2 mg (75%) of **16** as a white solid. ^1^H–NMR (400 MHz, DMSO-*d*_6_) *δ* 9.96 (*s*, 1H), 7.56 (*d*, *J* = 8.6 Hz, 2H), 7.28 (*d*, *J* = 8.6 Hz, 2H), 6.15 (*dd*, *J* = 15.0, 2.6 Hz, 2H), 4.30 (*d*, *J* = 3.6 Hz, 1H), 3.00 (*dd*, *J* = 13.8, 3.9 Hz, 1H), 2.35 (*dd*, *J* = 13.7, 6.2 Hz, 3H), 2.26 (*t*, *J* = 7.3 Hz, 2H), 1.80 (*p*, *J* = 7.3 Hz, 2H). ^13^C–NMR (101 MHz, DMSO-*d*_6_): *δ* 174.2, 170.8, 138.1, 137.4, 131.0, 128.3, 119.5, 46.1, 39.4, 35.4, 33.1, 20.5. HRMS [M+Na]^+^ *m/z* calcd. for [C_15_H_17_NNaO_3_S]^+^ 314.0821, found 314.0821.

#### 3.2.9. Synthesis of 2,5-Dioxopyrrolidin-1-yl-5-((4-(Cyclobut-2-en-1-Ylthio)Phenyl)Amino)-5-Oxopentanoate (**17**)

A mixture of compound **16** (6.2 mg, 0.021 mmol, 1.0 equiv), *N,N*-disuccinimidyl carbonate (DSC) (5.4 mg, 0.021 mmol, 1.0 equiv), and TEA (4.5 μL, 0.032 mmol, 1.5 equiv) was dissolved in tetrahydrofuran (THF) (0.5 mL) under Ar. The resulting mixture was stirred at room temperature for 4 h. After reaction completion, the reaction was quenched with water and extracted with ethyl acetate. The combined organic phases were washed with brine, dried over anhydrous Na_2_SO_4_, and evaporated in vacuo. Similarly to analogue **16**, the residue was purified by thin-layer chromatography to afford the desired product **17** as a white solid in 51% yield (4.2 mg). ^1^H–NMR (400 MHz, CDCl_3_) *δ* 8.02 (*s*, 1H), 7.47 (*d*, *J* = 8.5 Hz, 2H), 7.30 (*d*, *J* = 8.6 Hz, 2H), 6.09 (*dd*, *J* = 21.8, 2.5 Hz, 2H), 4.26 (*d*, *J* = 3.5 Hz, 1H), 3.02 (*dd*, *J* = 13.8, 3.6 Hz, 1H), 2.88 (*s*, 4H), 2.75–2.68 (*m*, 2H), 2.47 (*dd*, *J* = 12.9, 5.8 Hz, 3H), 2.23–2.12 (*m*, 2H). ^13^C–NMR (101 MHz, CDCl_3_) *δ* 170.2, 169.7, 168.5, 138.0, 137.6, 136.9, 131.7, 130.9, 120.3, 47.0, 40.0, 35.5, 30.0, 25.8, 21.2. HRMS [M+Na]^+^ *m/z* calcd. for [C_19_H_20_N_2_NaO_5_S]^+^ 411.0985, found 411.0989.

### 3.3. Stability of the Cyclobutene Derivatives **6a**–**6d**, **11a**, and **11b**

To examine the stability of the synthesized analogues for potential applications in bioorthogonal chemistry, dienophiles **6a–6d**, **11a**, and **11b** were dissolved in a DMF/PBS mixture (1:1) at a final concentration of 0.5 mM at pH 7.4 and 37 °C. Samples were monitored at 0, 12, and 24 h using high-performance liquid chromatography (3 μL injected per time point). Absorbance was measured at 260 nm for **6b**, 280 nm for **11a** and **11b**, 254 nm for **6a** and **6d**, and 300 nm for **6c**. The peak area at each time point was expressed as a percentage of the area at 0 h.

### 3.4. Kinetics of the Reactions between Cyclobutene Derivatives and Tetrazine

Tetrazine **12** was dissolved in a DMF/H_2_O (1:1) mixture at a final concentration of 0.5 mM at 37 °C. Dienophiles **6a–6d**, **11a**, and **11b** were then added to the quartz cuvettes with final concentration of 5 mM. A Quawell Q6000+ UV–Vis spectrophotometer was used to monitor the disappearance of the characteristic absorption peak of tetrazine at 520 nm at 10-min intervals for a duration of 6 h. The experimental data were processed using GraphPad Prism 6.0.

### 3.5. IEDDA-Oxidation Cascade Reaction of Cyclobutene **6a** with Dipyridinetetrazine **12**

Compound **6a** (5 mg, 0.028 mmol, 1.05 equiv) and dipyridine tetrazine **12** (6.38 mg, 0.027 mmol, 1.0 equiv) were dissolved in THF (0.3 mL) and H_2_O (0.1 mL). The resulting mixture was stirred at 60 °C for 2 h. Upon completion monitored by HPLC, to the reaction mixture was added DDQ (6.74 mg, 0.029 mmol, 1.1 equiv). The resulting mixture was stirred at room temperature for 1.5 h. Upon completion monitored by HPLC, the reaction was quenched with water and extracted with ethyl acetate. The combined organic phase was washed with brine, dried over anhydrous Na_2_SO_4_, and evaporated under reduced pressure. The residue was purified via silica column chromatography to afford the product **14** (8.5 mg, 82% yield) as a light red solid.

### 3.6. Protein Labeling

#### 3.6.1. Protein Modification In Vitro

The cyclobutene derivative **17** (0.5 μL, 50 mM, 10.0 equiv) was mixed with BSA (5 μL, 0.5 mM in PBS) in sodium bicarbonate buffer (39 μL, pH 8.2) containing DMSO (4.5 μL). The mixture was kept at room temperature for 2 h, then excess **17** was removed using a Zeba desalting spin column (0.5 mL). The modified protein was recovered in PBS and its concentration quantified at 19.2 μM based on Quawell Q6000+ UV–Vis absorption scanning.

#### 3.6.2. Protein Labeling In Vitro

Three samples were incubated at 37 °C for 4 h: the BSA-labeling reaction containing H-Tz-Cy5 (2.5 μL, 5 mM in DMSO) and cyclobutene-modified BSA (22.5 μL, 19.2 μM in PBS), giving a final dye concentration of 250 μM or 500 μM; a negative-control reaction containing H-Tz-Cy5 (2.5 μL, 5 mM in DMSO) and unmodified BSA (22.5 μL, 19.2 μM in PBS), again giving a final dye concentration of 250 μM or 500 μM; and a “blank” reaction of only neat, unmodified BSA (25 μL, 19.2 μM in PBS). After incubation, all of the samples were passed through a Zeba desalting spin column (0.5 mL) to remove excess dye. All of the column eluates contained the same concentration of about 0.4 mg/mL, based on Quawell Q6000+ spectrophotometry. An aliquot (20 μL) of each sample was mixed with 2 μL of 5X SDS–PAGE loading buffer and vortexed for 10 sec, and an aliquot (15 μL) of supernatant was loaded onto SDS–PAGE gels. The gels were imaged for fluorescence using the ChemiDoc™ MP imaging system (Bio-Rad), then stained with Coomassie blue and imaged under visible light.

#### 3.6.3. Rough Estimation Method of the Yield of the Labeling Reaction on the Model BSA Protein

The second-order reaction rate constant of compound **6d** and 3-phenyl-1,2,4,5-tetrazine (H-Tz-Ph) was determined by a method similar to that described above for determining reaction kinetics, giving *k*_2_ = 0.056 M^−1^s^−1^.

The first-order reaction rate constant (*k*_1_) was determined according to the formula:

(1) $$k_{1}=ck_{2}$$

*c* is the concentration of the reactant, which is greatly excessive.

The half-life (*t*_1/2_) of the reaction was calculated with the following formula:

(2) $$t_{1/2\;}=\;\frac{\ln2}{k_{1}}$$

The final dye concentration in the labeling reaction was 500 μM and the reaction time was 4 h. The half-life (*t*_1/2_) was calculated according to the above formulas and it was about 6.875 h. A rough estimation of the yield of the labeling reaction was about 29.1%, which is determined according to the formula:

(3) $$\mathrm{Yield}\;\left(4\;h\right)=\;\frac{4\;h}{t_{1/2}}\times 50\%$$

## 4. Conclusions

We developed concise synthesis of a series of novel aryl-substituted cyclobutene derivatives. These small, strained cyclobutenes are stable enough to serve as small dienophiles for bioorthogonal tetrazine ligations that proceed with good kinetics and high yields. Yet, the aryl-substituted cyclobutenes might not be small enough for use as mini-tags for bioorthogonal metabolic labeling applications and of relatively poor water solubility. Our synthesis strategy provides a potential way to synthesize hydrophilic alky-substituted cyclobutene. Based on these promising results, we expect that the synthesis of additional aryl-substituted cyclobutene analogues will significantly enrich available bioorthogonal tools and facilitate the development of new biomedical applications.

## Data Availability

Data sharing not applicable.

## Sample Availability

Not available.
